# Volatile MoS_2_ Memristors with Lateral Silver Ion Migration for Artificial Neuron Applications

**DOI:** 10.1002/smsc.202400523

**Published:** 2025-01-27

**Authors:** Sofía Cruces, Mohit Dineshkumar Ganeriwala, Jimin Lee, Lukas Völkel, Dennis Braun, Annika Grundmann, Ke Ran, Enrique González Marín, Holger Kalisch, Michael Heuken, Andrei Vescan, Joachim Mayer, Andrés Godoy, Alwin Daus, Max Christian Lemme

**Affiliations:** ^1^ Chair of Electronic Devices RWTH Aachen University Otto‐Blumenthal‐Str. 25 52074 Aachen Germany; ^2^ Department of Electronics and Computer Science Universidad de Granada Avenida de la Fuente Nueva S/N 18071 Granada Spain; ^3^ Compound Semiconductor Technology RWTH Aachen University Sommerfeldstr. 18 52074 Aachen Germany; ^4^ Central Facility for Electron Microscopy RWTH Aachen University Ahornstr. 55 52074 Aachen Germany; ^5^ Ernst Ruska‐Centre for Microscopy and Spectroscopy with Electrons (ER‐C 2) Forschungszentrum Jülich GmbH Wilhelm‐Johnen‐Str. 52425 Jülich Germany; ^6^ AMO GmbH Advanced Microelectronic Center Aachen Otto‐Blumenthal‐Str. 25 52074 Aachen Germany; ^7^ AIXTRON SE Dornkaulstr. 2 52134 Herzogenrath Germany; ^8^ Sensors Laboratory Department of Microsystems Engineering University of Freiburg Georges‐Köhler‐Allee 103 79110 Freiburg Germany

**Keywords:** 2D materials, compact model, ion migration, lateral memristor, neuromorphic computing, resistive switching

## Abstract

Layered 2D semiconductors have shown enhanced ion migration capabilities along their van der Waals (vdW) gaps and on their surfaces. This effect can be employed for resistive switching (RS) in devices for emerging memories, selectors, and neuromorphic computing. To date, all lateral molybdenum disulfide (MoS_2_)‐based volatile RS devices with silver (Ag) ion migration have been demonstrated using exfoliated, single‐crystal MoS_2_ flakes requiring a forming step to enable RS. Herein, present volatile RS with multilayer MoS_2_ grown by metal‐organic chemical vapor deposition (MOCVD) with repeatable forming‐free operation is presented. The devices show highly reproducible volatile RS with low operating voltages of ≈2 V and fast‐switching times down to 130 ns considering their micrometer‐scale dimensions. The switching mechanism is investigated based on Ag ion surface migration through transmission electron microscopy, electronic transport modeling, and density functional theory. Finally, a physics‐based compact model is developed and the implementation of the volatile memristors as artificial neurons in neuromorphic systems is exploredd.

## Graphical abstract

This article investigates lateral memristors made from metal‐organic chemical vapor deposition‐grown multilayer molybdenum disulfide, which exhibit repeatable, forming‐free resistive switching with low voltages and fast‐switching times. The switching mechanism based on silver ion surface migration is corroborated through electronic transport modeling and density functional theory calculations. The volatile memristors are implemented as artificial neurons in neuromorphic systems through compact modeling.

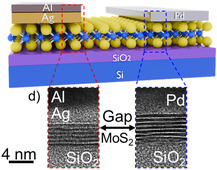

## 1 Introduction

2D layered materials, including semiconducting transition metal dichalcogenides (TMDCs), have long been known to allow ion intercalation and migration within their van der Waals (vdW) gaps and on their surfaces.^[^ ^1^, ^2^, ^3^, ^4^, ^5^, ^6^, ^7^, ^8^, ^9^, ^10^, ^11^, ^12^, ^13^ ^]^ More recently, these unique properties have been utilized to demonstrate applications such as emerging memory technologies, electronic switches (e.g., as selectors), and neuromorphic computing.^[^ ^8^, ^10^, ^14^, ^15^, ^16^, ^17^, ^18^, ^19^, ^20^, ^21^ ^]^ In certain device configurations, the number and type of defects and specific intrinsic properties of layered 2D materials (2DMs) have been shown to influence device performance and variability.^[^ ^13^, ^22^, ^23^, ^24^, ^25^, ^26^ ^]^ In the case of resistive switching (RS), the presence of defects such as cracks, lattice deformations, and dopants can positively impact RS, for example, lower the switching voltages.^[^ ^27^, ^28^ ^]^ This is in contrast to 2DM‐based field effect transistors, which require nearly defect‐free 2DMs to ensure high charge carrier mobility, low charge trap density, structural stability, and resistance to degradation over time.^[^ ^28^, ^29^ ^]^ Hence, understanding the growth of large‐scale layered 2DMs is important for delivering application‐specific materials for future integration into semiconductor fabrication lines.^[^ ^27^, ^28^, ^29^, ^30^ ^]^

Lateral devices for RS applications have three main advantages when compared to vertical stacks: 1) the selector functionality can be included; 2) the signal transmission and learning function can be performed simultaneously; 3) they offer spatiotemporal information processing.^[^ ^31^, ^32^ ^]^ Previously, RS devices based on lateral ion migration on smooth molybdenum disulfide (MoS_2_) surfaces have been reported.^[^ ^7^, ^10^, ^12^, ^17^, ^33^, ^34^, ^35^ ^]^ However, these approaches are limited to nonscalable device configurations employing mechanically exfoliated MoS_2_ or single‐crystal MoS_2_ flakes and face scalability, uniformity, and variability issues.^[^ ^7^, ^10^, ^12^, ^17^, ^33^ ^]^ In addition, metal‐organic chemical vapor deposition (MOCVD) or chemical vapor deposition (CVD) have been used to grow large‐area 2DM films.^[^ ^31^, ^36^, ^37^ ^]^ Thus far, lateral devices with memristive behavior induced by vacancy or grain boundary migration in CVD‐grown monolayer MoS_2_ have been studied. However, the voltages applied to switch these devices to their on‐state were between 3.5 and 20 V, which is too high for low‐power applications, such as neuromorphic computing.^[^ ^38^, ^39^ ^]^ Moreover, these lateral MoS_2_ devices required “forming” to initiate RS, that is, the application of large voltages and currents to induce the first switching event. These factors can induce damage or lead to high device‐to‐device variability.^[^ ^20^, ^40^ ^]^ In addition, neuromorphic computing concepts typically involve integration with advanced complementary metal oxide semiconductor (CMOS) circuits, which cannot easily provide such high voltages. Moreover, the potential of lateral 2DM‐based resistive switches as artificial neurons remains largely unexplored.^[^ ^6^, ^18^ ^]^

Here, we study volatile 2DM‐based lateral memristive devices operating through silver (Ag) ion migration with low operating voltages and high reproducibility, including their potential as artificial neurons. We demonstrate forming‐free, volatile RS in micron‐sized memristive devices fabricated with multilayer MoS_2_ grown by MOCVD.^[^ ^41^, ^42^, ^43^ ^]^ We explore the current–voltage (*I*–*V*) characteristics and switching dynamics of devices with lateral distances between electrodes (gaps) down to ≈1 μm. Furthermore, we present highly reproducible volatile RS with over several hundred cycles, fast switching down to 130 ns, and low switching voltages of ≈2 V, which is a notable advancement for micron‐sized lateral 2DM‐based memristive devices.^[^ ^38^, ^39^, ^44^ ^]^ The experimental realization is complemented with a physics‐based model supported by ab‐initio simulations. The model connects the migration dynamics of Ag ions with electron transport along our palladium (Pd)/MoS_2_/Ag structures. Finally, we use the model to show the potential operation of our MoS_2_ volatile memristors as artificial neurons in neuromorphic systems.

## 2 Results and Discussion

**Figure 1:**
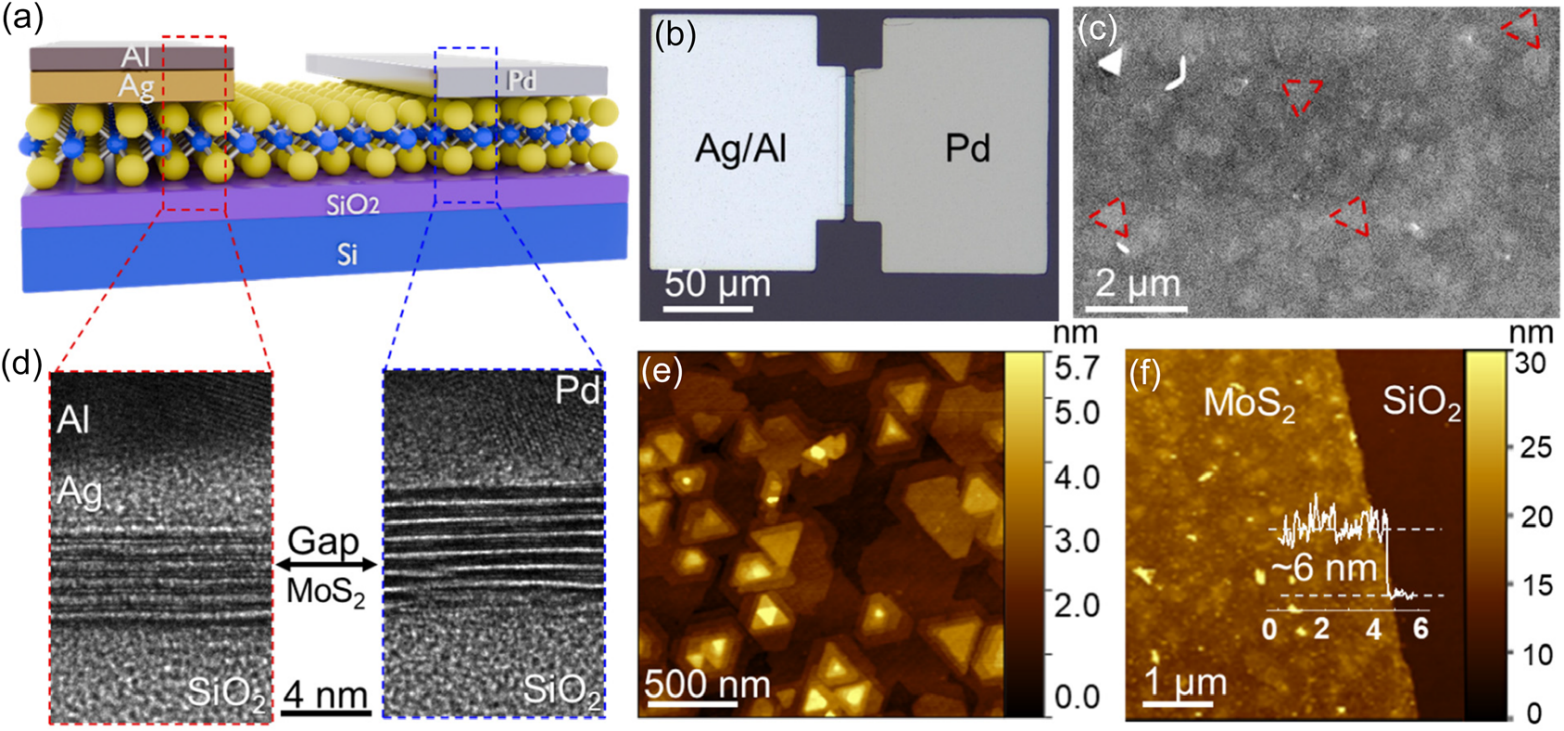
Cross‐section of the device structure and material characterization data. a) Schematic and b) optical microscopy image of our Al/Ag/MoS_2_/Pd device. c) Top‐view SEM image of the crystalline structure of MoS_2_ after device fabrication. The white triangles (marked in red) indicate possible multilayer nucleation sites. d) TEM images of both electrodes and MoS_2_. e) AFM image of MOCVD MoS_2_ as grown on 2” sapphire. f) AFM image of MoS_2_ after transfer to SiO_2_/Si. The inset height profile shows a thickness of ≈6 nm.

The devices investigated in this work consisted of multiple layers of MOCVD‐grown MoS_2_ with one Pd and one Ag electrical contact. The Ag ions can migrate on the surface of the MoS_2_ layers and modulate the channel conductivity, driven by a lateral electrical field. **Figure** **1**a shows a schematic of such a lateral MoS_2_ memristive device.

MoS_2_ was grown via MOCVD on 2” sapphire and wet transferred onto a SiO_2_/Si substrate (see Experimental Section). The metal contacts were deposited by electron‐beam (e‐beam) evaporation of 50 nm Pd and 50 nm Ag. An additional 50 nm aluminum (Al) layer was evaporated on top of the Ag contacts to avoid Ag tarnishing^[^ ^45^ ^]^ (see Figure S1, Supporting Information). The MoS_2_ channels in the gap between contacts were patterned via reactive ion etching (RIE), and afterwards, the complete removal of MoS_2_ next to the channels, was verified via Raman mapping (Figure S2, Supporting Information). Figure 1b shows a top‐view optical microscopy image of a fabricated device with a gap size of 5 μm. The experiments included devices with gaps between ≈1 and ≈6 μm. Figure 1c displays a top‐view scanning electron microscopy (SEM) image of the polycrystalline structure of MoS_2_ after device fabrication. Two different features can be distinguished: white triangles (marked in red), which are probable multilayer nucleation sites, and brighter white features, which may be vertically aligned sheets from the growth process. The images do not show photoresist residues on the MoS_2_ surface from the fabrication process. High‐resolution transmission electron microscopy (HRTEM) cross‐section images of the Pd and Ag/Al top contacts on MoS_2_ are presented in Figure 1d. The layered 2D structure of the multilayer MoS_2_ film can be clearly recognized, with an interlayer distance of 6.2 ± 0.05 Å, which coincides with reported values in the literature.^[^ ^11^, ^46^ ^]^ Figure 1e presents an atomic force microscopy (AFM) image of the polycrystalline, as‐grown MoS_2_ on 2” sapphire. Figure 1f depicts the morphology of the material after transfer, and the inset height profile indicates a thickness of ≈6 nm. HRTEM cross‐sectional images at two different positions on the Pd electrode side revealed slight thickness variations in the MoS_2_ of approximately five layers (Figure S3, Supporting Information). In addition, we performed Raman analysis of the as‐grown material on sapphire and after transfer. The measurements were not taken at the exact same location, which could lead to a slight frequency difference due to thickness variation in the sample.^[^ ^47^ ^]^ The extracted E^1^_2g_ and A_1g_ peaks of MoS_2_ coincide with the literature values for more than four layers or the bulk, which is in agreement with the AFM and HRTEM data (see Figure S4, Supporting Information).^[^ ^47^, ^48^ ^]^

**Figure 2:**
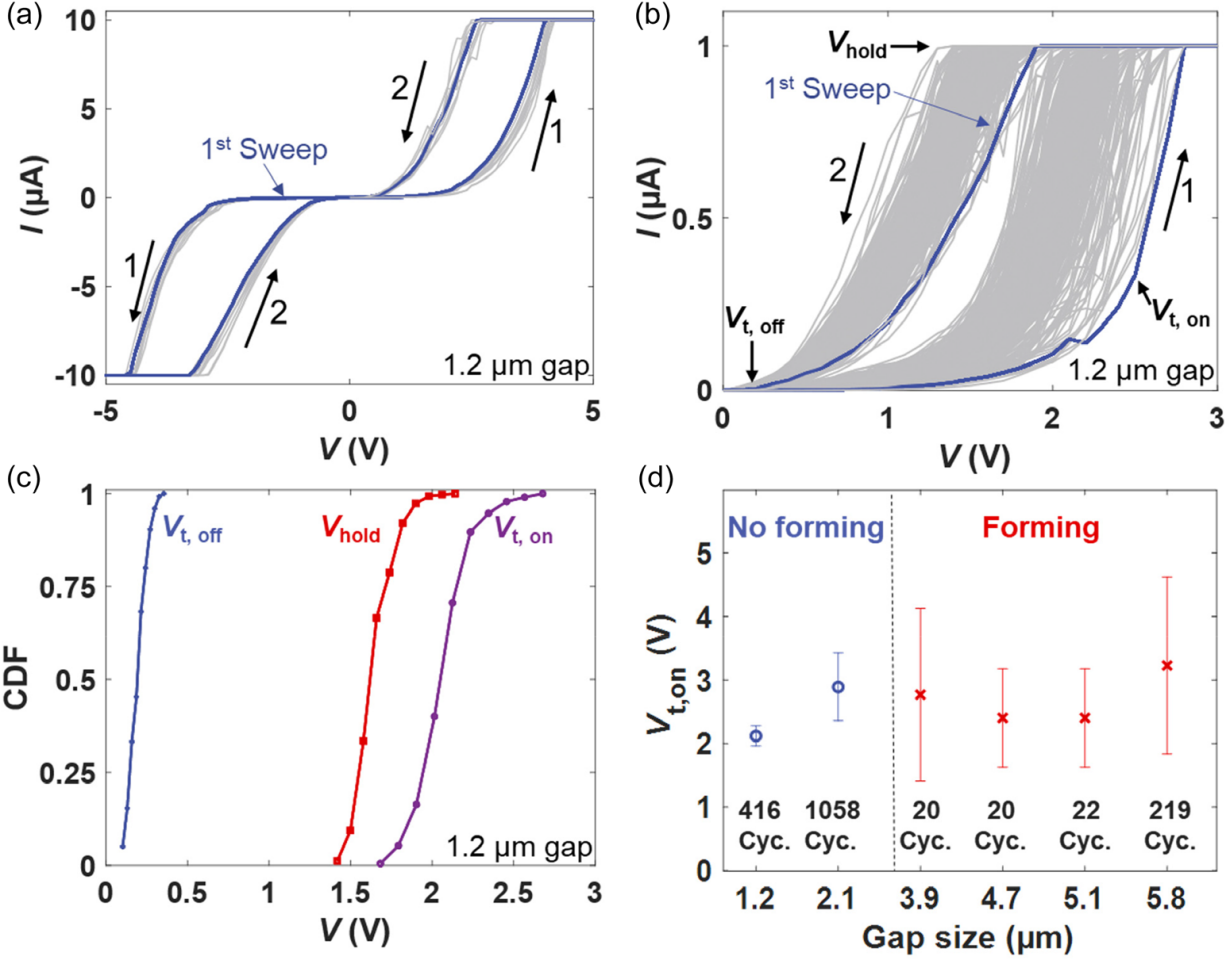
DC *I*‐*V* characterization of Pd/MoS_2_/Ag memristive devices. a) 10 *I–V* curves recorded in both voltage polarities. Arrows 1 and 2 show the voltage sweep direction. The first sweep is marked in blue. b) Over 400 consecutive switching cycles from a 1.2 μm gap device. The characteristic voltages are marked on the curves. c) Cumulative distribution function of *V*_t,on_, *V*_t,off_ and *V*_hold_ of the data displayed in b). (d) Dependence of *V*_t,on_ on the gap size for different devices with different numbers of subsequent RS cycles.

We performed *I*–*V* measurements of the lateral MoS_2_‐based memristors by applying a voltage to the Ag electrode while the Pd electrode and the back gate were grounded. **Figure** **2**a presents ten subsequent voltage sweeps in both positive and negative polarities that show similar volatile RS behavior. The *I*–*V* sweeps were conducted first in the positive direction from 0 to 5 V (arrow number 1) and back to 0 V (arrow number 2), followed by the same procedure in the negative direction. This first switching cycle is marked in blue to show that the initial RS required no forming event at higher voltages. In addition, first switching cycles of several forming‐free devices are shown in Figure S5, Supporting Information. This behavior was specifically observed for devices with gap sizes ranging between ≈1 and ≈2 μm. Figure 2b displays over 416 switching cycles on a device with a gap size of ≈1.2 μm. For a better visibility of the low currents, the *I*–*V* curves are displayed in logarithmic scale in Figure S6, Supporting Information. Initially, the device is in a high‐resistance state (HRS) during the forward sweep until the transition to a low‐resistance state (LRS) occurs at an “on‐threshold” voltage (*V*_t,on_) of ≈2.1 V. For this measurement, the current compliance (CC) was set to 1 μA. The initial resistance states for different devices are displayed in Figure S7, Supporting Information. Moreover, Figure S8, Supporting Information displays the forming *I*–*V* curves of those devices that required forming in Figure S7, Supporting Information. The device remained in the LRS during the backward sweep until it switched back below a “hold” voltage (*V*_hold_) of ≈1.7 V. The original HRS was reached at an “off‐threshold” voltage (*V*_t,off_) of ≈0.2 V. Moreover, we observed volatile RS over several cycles at a comparatively higher CC (see Figure S9, Supporting Information).

Furthermore, we conducted *I*–*V* sweeps on four devices with different metal combinations with and without MoS_2_ to support our hypothesis that the RS originates from Ag ion migration on or between the MoS_2_ layers. We observed volatile RS only for the Pd/MoS_2_/Ag device (see Figure S10, Supporting Information).

We plotted *V*_t,on_, *V*_t,off_, and *V*_hold_ of the 416 switching cycles in a histogram as an indirect measure of endurance and fitted the data with a Gaussian distribution (Figure S11, Supporting Information). The statistical distribution of the characteristic voltages shows reasonably low standard deviations (*σ*) of ≈0.1 V and low cycle‐to‐cycle variabilities of *V*_t,on_ and *V*_hold_ of 4.76 and 5.95%, respectively. Figure 2c presents the normalized cumulative distribution functions (CDFs) of *V*_t,on_, *V*_hold_, and *V*_t,off_ of the 416 switching cycles, confirming the low variability among them. We attribute this low variability to the polycrystalline nature, thickness variations, and specific topography of our MOCVD MoS_2_ layers, in combination with our micron‐sized devices.^[^ ^38^, ^39^ ^]^ Since the numbers of grain sizes and thickness variations are high in each device, the variability in current transport is reduced because the individual differences in the resistance average out.^[^ ^49^ ^]^ The dependence of *V*_t,on_ upon the gap size between electrodes is plotted in Figure 2d, with all subsequent *I*–*V* sweeps for each device displayed in Figure S12, Supporting Information. The devices with gap sizes under 2.1 μm did not require a forming step, as previously mentioned. Devices with gap sizes of 3.9 μm and above, in contrast, required larger initial voltages between 10 and 50 V, depending on the gap size, to exhibit RS behavior. An extended plot of the dependence of *V*_t,on_ upon the gap size for both with forming and forming‐free devices is shown in Figure S13, Supporting Information.

The forming‐free switching of the 1 and 2 μm devices is strikingly different from that in prior works using single–crystal exfoliated MoS_2_, where forming was always necessary, and switching occurred only in submicron gaps.^[^ ^7^, ^12^ ^]^ Additionally, as illustrated in Figure 2d, the mean *V*_t,on_ for the device with a 1.2 μm gap size is ≈2.1 V. This value is mostly lower than or at least equal to the reported *V*_t,on_ values for devices with similar or shorter gap sizes.^[^ ^7^, ^33^, ^38^, ^39^, ^50^, ^51^ ^]^ A benchmarking table with more parameters is available in Table S1, Supporting Information.

**Figure 3:**
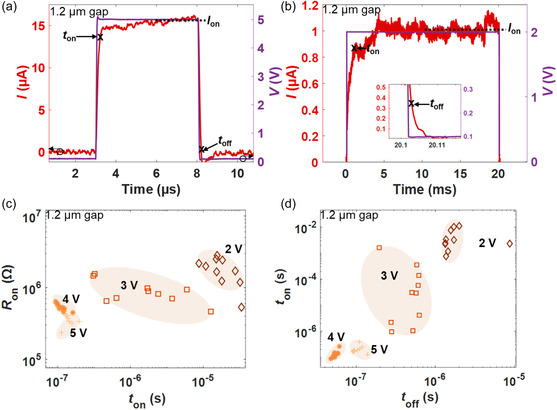
Dynamic response of threshold switching in an ≈1.2 μm gap size device. a) Pulsed waveform at a 5 V pulse amplitude showing switching on the order of 400 ns. b) Pulsed waveform at a 2 V pulse amplitude showing volatile switching on the order of 1.2 ms. c) Cycle‐to‐cycle variability of the ON‐state resistance (*R*_on_) and switching time (*t*_on_) for 10 sequentially pulsed waveforms at different pulse amplitudes. The device has a larger *t*_on_ with decreasing voltage pulse amplitude. d) Correlation of *t*_on_ and recovery time (*t*_off_) at different pulse amplitudes with varying pulse width at which RS was observed.

We analyzed the switching dynamics to characterize the transient behavior of our devices. Thus, we studied the time‐dependent current–response of the devices upon application of voltage pulses with various amplitude and timing parameters.^[^ ^52^, ^53^ ^]^ The switching time (*t*_on_) was defined as the time needed to reach 90% of the ON current (*I*_on_), and the recovery time (*t*_off_) was defined as the time required to reach 10% of the difference between *I*_on_ and the OFF current (*I*_off_) when switching back to the HRS. More information about how the switching parameters were extracted can be found in Figure S14, Supporting Information. **Figure** **3**a displays a typical time‐dependent response upon the application of a 5 V pulse with a duration of 5 μs to a device with a 1.2 μm gap size. We obtained characteristic response times, such as *t*_on_ and *t*_off_, of <400 and <150 ns, respectively. *t*_on_ and *t*_off_ are crucial parameters for memristive device operation. Both switching parameters can be influenced by external factors such as the voltage pulse amplitude and the width of the pulsed waveform. For example, with the application of a voltage pulse of 2 V and length of 20 ms, the extracted values for *t*_on_ and *t*_off_ drastically change to 1.2 ms and 1.45 μs, respectively (Figure 3b). Furthermore, we applied ten consecutive pulses with varying pulse widths and increasing voltage amplitudes to extract the ON‐state resistance (*R*_on_), *t*_on_ and *t*_off_ for each switching cycle. Table S2, Supporting Information summarizes the pulse programming parameters and is available in the Supporting Information. Figure 3c shows how *R*_on_ decreases with increasing voltage pulse amplitude. The increasing voltage leads to greater Ag ion diffusion, which decreases the average distance between the ions and increases the current (see model in Section S18, Supporting Information). We analyzed the switching dynamics of *t*_on_ and *t*_off_ in more detail (Figure 3d) and found that *t*_on_ decreases exponentially with increasing voltage pulse amplitude. This behavior is consistent with other ion diffusion‐based memristive devices.^[^ ^54^, ^55^, ^56^ ^]^ Moreover, for the 4 and 5 V pulse amplitudes, *t*_on_ displays the lowest variability and the fastest switching times. It has been shown in the literature that nanoscale 2DM‐based memristive devices usually have switching times in the nanosecond range (30–200 ns).^[^ ^14^, ^19^, ^24^ ^]^ Here, we observe such fast switching in lateral MoS_2_‐based memristive devices with micron‐sized gaps. We attribute this behavior to the presence of grain boundaries in our polycrystalline MoS_2_, which may facilitate Ag ion migration in the lateral structures.^[^ ^38^, ^39^, ^57^ ^]^ After several *I–V* sweeps, Ag ions are distributed across the MoS_2_ channel, thereby reducing the effective distance between electrodes and resulting in faster switching times.^[^ ^12^, ^27^, ^38^, ^57^ ^]^ The mean *t*_on_ of our micron‐sized gap devices for 4 and 5 V ranges from 130 up to 200 ns. Such fast‐switching performance would be beneficial for selector and neuromorphic computing applications.^[^ ^54^, ^55^, ^58^, ^59^ ^]^ In the case of 2 and 3 V pulses, the device shows higher cycle‐to‐cycle variability and longer switching times ranging from 0.2 up to 4.5 ms. In addition, the devices required longer pulses in the millisecond range to observe the RS. Nevertheless, slower switching times at low voltages could be advantageous for some applications. For example, recent works have shown that threshold memristors with low voltages and long switching times are a natural choice for the realization of artificial neurons.^[^ ^6^, ^10^, ^18^ ^]^

**Figure 4:**
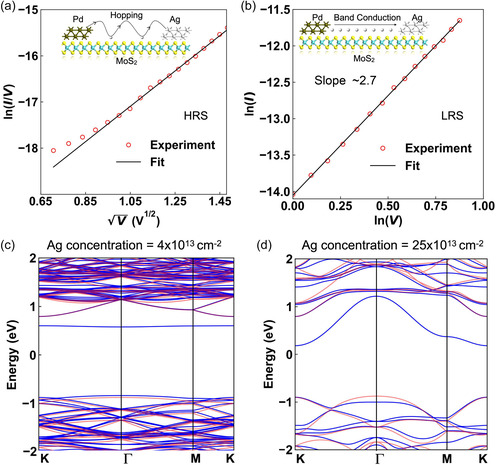
*I*–*V* characteristics of the volatile memristor. a) HRS shows Poole–Frenkel‐type hopping transport, while b) LRS shows space‐charge limited conduction (SCLC). Band structure of MoS_2_ calculated via density functional theory (DFT) (blue line) on a highly symmetric path along the 2D Brillouin zone for two different surface‐adsorbed Ag concentrations: c) 4 × 10^13^ cm^−2^ and d) 25 × 10^13^ cm^−2^, respectively. The band structure of pristine MoS_2_ is also included in red for comparison. Note that the band structure is shifted to coincide with the conduction band of pristine MoS_2_.

We analyzed the current conduction mechanisms in the HRS and LRS using the median of the *I*–*V* cycles shown in Figure 2a and found different transport mechanisms in the HRS and LRS. In the HRS (i.e., for forward sweep voltages ranging up to ≈*V*_t,on_), the device shows a linear relation between ln(*I*/*V*) and sqrt(*V*) (**Figure** **4**a). Such a dependency is indicative of hopping transport through localized states (such as Poole–Frenkel (PF) hopping), where the mobility is increased due to the applied electric field.^[^ ^60^, ^61^, ^62^ ^]^ In the LRS, in contrast, the *I*–*V* characteristics best match the space‐charge‐limited conduction (SCLC) mechanism (Figure 4b). In particular, the ln(*I*) versus ln(*V*) plot (for the backward sweep starting at the CC down to voltages between *V*_hold_ and *V*_t,off_) shows a slope of ≈2.7, indicating that the current in the LRS follows the Mott–Gurney law of SCLC with PF‐enhanced mobility ($I\propto V^{2+m},\;m\geq 0$).^[^ ^63^ ^]^ The current conduction mechanism, therefore, changes from PF hopping to SCLC during the voltage sweep of memristive devices. This switching can be attributed to the movement of Ag ions along the MoS_2_ surface, which have been shown to be highly active and responsible for RS in previous investigations^[^ ^7^, ^12^, ^54^ ^]^ (see insets in Figure 4a,b). The dependence of *t*_on_ on the applied pulse amplitude (Figure 3d) also points toward the involvement of ionic movement in the RS.

We carried out first‐principles simulations using density functional theory (DFT) to analyze the electronic properties of MoS_2_ in the presence of Ag atoms (see the Experimental Section for more details). In particular, Figure 4c shows the calculated band structure of monolayer MoS_2_ with a low concentration of Ag atoms adsorbed on the surface on a highly symmetric path along the Brillouin zone (marked in blue), together with the band structure of a pristine MoS_2_ layer (marked in red) for comparison. The adsorption of the Ag atom on the MoS_2_ surface gives rise to a state in the bandgap close to the conduction band. However, the near dispersion‐less nature of this state indicates that it is spatially localized. Such a state could facilitate the hopping of a carrier through the otherwise semiconducting MoS_2_, as depicted schematically in the inset of Figure 4a. Increasing the surface concentration of Ag atoms reduces their average distance, resembling the formation of a conductive filament and creating a noticeable change in the Ag energy states (Figure 4d). The localized states are thus transformed into a delocalized band, which supports band‐like transport. The intermediate stages of this transformation correspond to the gradual increase in the Ag concentration and the associated transformation of the band gap state from a localized to a delocalized band is detailed in Figure S15, Supporting Information.

On the basis of these results, the RS mechanism can be understood as follows: initially, the memristor is in the HRS. The application of a voltage bias then results in the drift of active Ag ions across the MoS_2_ surface, causing carrier transport by PF hopping through the localized, Ag ion‐induced states. The polycrystalline MoS_2_, which was used in this work, may also exhibit similar PF hopping conduction, even when the Ag concentration is negligible.^[^ ^64^ ^]^ Increasing the voltage bias increases the Ag ion concentration, which modifies the band structure of the material and enables band‐like transport, leading to higher current levels in the LRS. It is important to note that the mechanism is not dependent on the specific species of Ag involved, as the formation of extended clusters of Ag nanoparticles or silver sulfide (Ag_2_S) is also possible. These clusters could provide hopping sites for electrons across the devices in the HRS. The movement of these clusters under the influence of an external electric field will eventually increase their concentration. The model suggests that even in the absence of a continuous conductive filament, the average distance between them will decrease if the concentration of Ag ions or clusters is sufficiently high, leading to the formation of a delocalized conduction band, which alters the transport mechanism. The volatile nature of the RS and the non‐ohmic *I*–*V* relationship in the LRS in our devices can be explained by the formation of a discontinuous Ag filament, which self‐ruptures when the voltage bias is reduced below *V*_hold_ as the Ag ions diffuse away, resetting the device back to the HRS.^[^ ^54^ ^]^

**Figure 5:**
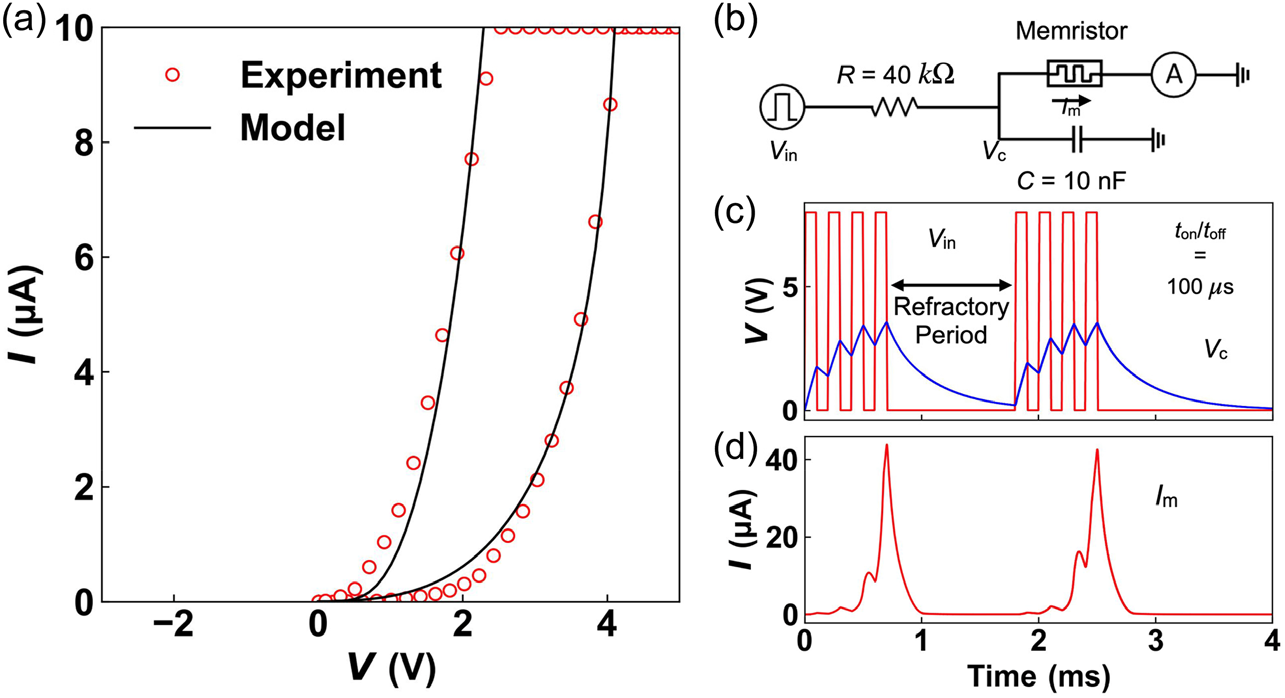
a) Experimental *I–V* characteristics of the experimental memristor (symbols) matched with the developed model (solid lines), showing good agreement. b) Circuit implementation of the leaky integrate‐and‐fire model of the neuron using the memristor. SPICE simulation results showing c) input pulse train (*V*_in_) with *t*_on_ and *t*_off_ = 100 μs and a refractory period of 1000 μs along with the voltage across the capacitor (*V*_c_) and d) current through the memristor (*I*_m_) showing high current spikes at the end of the integration period, emulating the firing of the neuron.

Our volatile lateral MoS_2_ memristors, with their dynamic thresholding, can be harnessed to implement artificial neurons in neuromorphic computing systems.^[^ ^6^, ^65^ ^]^ To demonstrate this possibility, we developed a physics‐based model capturing the observed Ag dynamics that give rise to the RS, as well as the electronic current conduction mechanisms in both resistive states. Details of the model and its equations are summarized in Section S18, Supporting Information. **Figure** **5** shows the *I*–*V* model, which phenomenologically captures the experimental dynamics of the volatile memristors to a high degree. This enables us to emulate the experimental behavior of the memristor in circuit‐level simulations. We implemented the model in Verilog–A and investigated the leaky integrate‐and‐fire (LIF) model of the neuron shown in Figure 5b. Here, the capacitor (*C*), along with the parallel memristor, operates as an LIF neuron's soma, whereas the series resistance (*R*) acts as the synaptic weight. The SPICE simulation results (Figure 5c) show the input pulse voltage (*V*_in_) emulating the spike signals arising from the presynaptic neurons. Since the parallel memristor is initially in its HRS, the capacitor integrates the input signal into its membrane potential (*V*_c_). When the potential reaches the threshold voltage of the neuron (≈*V*_t,on_), the memristor switches to the LRS, resulting in a sudden current spike (Figure 5d) equivalent to the firing of a neuron. The LRS of the memristor enables the discharging of the capacitor, mimicking leaky neuron behavior, and *V*_c_ reaches its original potential after some period of inactivity of the presynaptic neuron, as shown in Figure 5d. After firing, the cell must wait for the so‐called refractory period before it can begin a new integration and firing process.^[^ ^66^ ^]^ Our circuit‐level simulations demonstrate the suitability of the fabricated MoS_2_ lateral memristors as artificial neurons, confirming their potential for application in future neuromorphic systems.

## 3 Conclusion

We demonstrated forming‐free, volatile RS in memristive devices with micrometer‐sized gaps fabricated from multilayer polycrystalline MoS_2_. Our devices exhibit highly reproducible volatile RS with over several hundred cycles, switching voltages of ≈2 V, and fast switching within a few hundred nanoseconds for 5 V pulses. We analyzed the current conduction mechanisms for both the HRS and LRS and concluded that Poole–Frenkel hopping and space‐charge limited conduction are dominant, respectively. In addition, we captured the switching dynamics of Ag ions and explained the electron transport along our experimental lateral Pd/MoS_2_/Ag–Al memristors with a physics‐based model. Finally, the model was employed to demonstrate the potential of our volatile memristors for artificial neurons in neuromorphic systems.

## 4 Experimental Section

4.1

4.1.1

##### Metal–organic Chemical Vapor Deposition (MOCVD) of MoS_2_

Highly uniform MoS_2_ was epitaxially grown in a commercial AIXTRON planetary reactor in a 10 × 2” configuration on sapphire (0001) substrates. First, the substrate was prebaked at 1050 °C in a pure H_2_ atmosphere to promote lateral growth by preventing the formation of a parasitic carbonaceous film.^[^ ^41^ ^]^ The growth process was carried out at a substrate temperature of 845 °C, with nitrogen as the carrier gas and a pressure of 30 hPa. A high sulfur to molybdenum ratio of 200 000 was chosen to achieve homogeneous MoS_2_ films on a wafer scale. To achieve this, the precursor flow rate was set to 0.1 nmol min^−1^ for molybdenum hexacarbonyl (MCO) and 20 μmol min^−1^ for di‐tert‐butyl sulfide (DTBS).^[^ ^42^ ^]^ A schematic of the MOCVD growth process is displayed in Figure S17, Supporting Information.

##### Device Fabrication

MoS_2_ grown on 2” sapphire by MOCVD was transferred onto 2 × 2 cm^2^ Si chips covered with 275 nm thermal SiO_2_. Poly(methyl methacrylate) (PMMA) was spin‐coated on top of the MoS_2_ before being released from the sapphire substrate in a potassium hydroxide (KOH) solution.^[^ ^42^ ^]^ The electrodes were defined with the AZ5214E JP photoresist from Merck Performance Materials GmbH and optical contact lithography with an EVG 420 Mask Aligner. The asymmetric Pd (50 nm) and Ag (50 nm)/Al (50 nm) electrodes were deposited via electron‐beam evaporation in a Pfeiffer tool and subsequently lifted in acetone at room temperature. Finally, the channels were patterned via CF_4_/O_2_ reactive ion etching (RIE) in an Oxford Instruments Plasma Lab System 100 tool. A flowchart outlining the main steps involved in the fabrication process is shown in Figure S18, Supporting Information.

##### Material and Device Characterization

Optical microscope images were recorded with a Leica INM100 microscope and a Keyence Laser Scanning microscope. Raman measurements were performed with a WiTec alpha300R Raman spectrometer in mapping mode with an excitation laser wavelength of 532 nm and 1 mW laser power. Scanning electron microscopy (SEM) images were taken with a Zeiss Supra 60VP SEM at an operation voltage of 4 kV. Transmission electron microscopy (TEM) analysis was conducted with a JEOL JEM F200 instrument at 200 kV. The lamella was prepared via a focused ion beam (FIB) with an FEI Strata400 system with a gallium (Ga) ion beam. Atomic force microscopy (AFM) measurements were conducted with a Dimension Icon AFM from Bruker Instruments in tapping mode.

##### Electrical Measurements

Electrical measurements were performed in a LakeShore probe station connected to a semiconductor parameter analyzer (SPA) “4200A–SCS” with two source measure unit (SMU) cards “Keithley 4200‐SMU”, each connected to a preamplifier “Keithley 4200‐PA” from Tektronix. A voltage was applied to the Ag/Al electrode, and the Pd electrode was grounded. Current–voltage (*I–V*) measurements were conducted by sweeping the voltage from 0 V to a positive maximum voltage *V*_max_ and back to 0 V. Cycling measurements were performed with a 0 s delay between each sweep cycle in normal mode. The current is limited by an external current limiter within the semiconductor parameter analyzer. Pulse experiments were performed by supplying a voltage to the Ag/Al electrode (channel 1) and measuring the output current over time in channel 2 (Pd electrode).

##### Theoretical Calculations

Density functional theory (DFT) calculations were carried out via the generalized gradient approximation (GGA) as implemented in QuantumATK with a linear combination of atomic orbitals (LCAO) basis set using the Perdew–Berke–Erzenhof exchange–correlation functional.^[^ ^67^, ^68^ ^]^ An SG15 pseudopotential with a medium‐size basis set was selected for the simulations.^[^ ^68^ ^]^ A vacuum layer of 20 Å was fixed along the out‐of‐plane direction to avoid interactions with periodic replicas of the system. First, a 5 × 5 supercell with a single Ag atom (corresponding to a concentration of 4 × 10^13^ cm^−2^) was considered. To further increase the Ag concentration, the supercell size was gradually reduced from 5 × 5 to 2 × 2. The Brillouin‐zone integration was performed over a Monkhorst–Pack grid of k‐points with a density (Å) of 4 × 4 × 1. A sufficiently large energy cutoff of 150 Ry was considered. The van der Waals (vdW) interactions were considered in the calculations through Grimme's DFT‐D2 dispersion corrections. The geometries and lattice parameters were fully relaxed until the forces acting on each atom were less than 0.01 eV Å^−1^. The integrity of the simulations was first verified by comparing the band structure of the pristine monolayer MoS_2_ and the obtained DFT‐based band gap of ≈1.7 eV with the literature.^[^ ^69^ ^]^

## Conflict of Interest

The authors declare no conflict of interest.

## Author Contributions

**Sofía Cruces**: conceptualization (supporting); formal analysis (lead); investigation (lead); methodology (equal); project administration (supporting); visualization (lead); writing—original draft (lead); writing—review & editing (lead). **Mohit D. Ganeriwala**: investigation (lead); software (equal); writing—original draft (supporting); writing—review & editing (supporting). **Jimin Lee**: formal analysis (supporting); writing—review & editing (supporting). **Lukas Völkel**: writing—review & editing (supporting). **Dennis Braun**: formal analysis (supporting); writing—review & editing (supporting). **Annika Grundmann**: resources (supporting). **Ke Ran**: data curation (supporting); investigation (lead); methodology (equal); writing—review & editing (supporting). **Enrique G. Marín**: software (supporting); supervision (supporting); writing—review & editing (supporting). **Holger Kalisch**: resources (supporting); writing—review & editing (supporting). **Michael Heuken**: funding acquisition (equal); resources (supporting); writing—review & editing (supporting). **Andrei Vescan**: funding acquisition (equal); resources (equal); supervision (equal); writing—review & editing (supporting). **Joachim Mayer**: funding acquisition (equal); resources (equal); supervision (equal); writing—review & editing (supporting). **Andrés Godoy**: funding acquisition (equal); resources (equal); software (supporting); writing—review & editing (supporting). **Alwin Daus**: conceptualization (equal); formal analysis (lead); investigation (equal); supervision (lead); writing—original draft (supporting); writing—review & editing (equal). **Max C. Lemme**: conceptualization (lead); formal analysis (equal); funding acquisition (lead); investigation (equal); project administration (lead); resources (lead); supervision (lead); writing—review & editing (lead).

## Supporting information

Supplementary Material

## Data Availability

The data that support the findings of this study are available from the corresponding author upon reasonable request.
