# Supplementary material for: Volatile MoS2 Memristors with Lateral Silver Ion Migration for Artificial Neuron Applications
Source: Small Sci. 2025 Jan 27;5(5):2400523. doi: 10.1002/smsc.202400523 (PMC12087773; doi:10.1002/smsc.202400523)
Supplement: Supplementary file 1 — Supplementary Material [file SMSC-5-2400523-s001.pdf]

Supporting Information

Volatile MoS_2 Memristors with Lateral Silver Ion Migration for Artificial Neuron Applications

Sofia Cruces[1], Mohit D. Ganeriwala[2], Jimin Lee[1], Lukas Völkel[1], Dennis Braun[1], Annika Grundmann[3], Ke Ran[4,5,8], Enrique G. Marín[2], Holger Kalisch[3], Michael Heuken[3,6], Andrei Vescan[3], Joachim Mayer[4,5], Andrés Godoy[2], Alwin Daus[1,7,*] and Max C. Lemme[1,8*].

[1] Chair of Electronic Devices, RWTH Aachen University, Otto-Blumenthal-Str. 25, 52074 Aachen, Germany

[2] Department of Electronics and Computer Science, Universidad de Granada, Avenida de la Fuente Nueva S/N 18071, Granada, Spain

[3] Compound Semiconductor Technology, RWTH Aachen University, Sommerfeldstr. 18, 52074 Aachen, Germany

[4] Central Facility for Electron Microscopy, RWTH Aachen University, Ahornstr. 55, 52074, Aachen, Germany

[5] Ernst Ruska-Centre for Microscopy and Spectroscopy with Electrons (ER-C 2), Forschungszentrum Jülich GmbH, Wilhelm-Johnen-Str., 52425 Jülich, Germany

[6] AIXTRON SE, Dornkaulstr. 2, 52134 Herzogenrath, Germany

[7] Sensors Laboratory, Department of Microsystems Engineering, Georges-Köhler-Allee 103, 79110 Freiburg, Germany

[8] AMO GmbH, Advanced Microelectronic Center Aachen, Otto-Blumenthal-Str. 25, 52074 Aachen, Germany

Section S1: Proof of silver (Ag) tarnishing

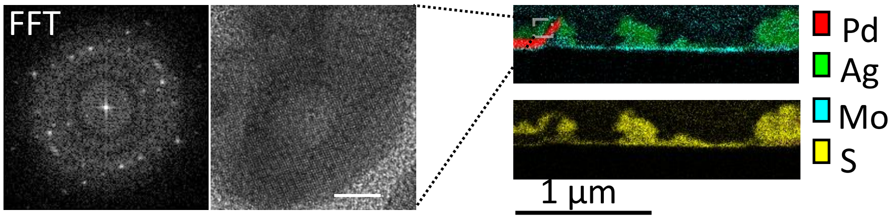

Figure S1: Proof of Ag tarnishing on a device after multiple electrical measurements. Based on the energy-dispersive x-ray spectroscopy (EDX) elemental mapping, both Ag and sulfur (S) are detected simultaneously between the palladium (Pd) and Ag metal contacts. Close to the Pd electrode region, high resolution transmission electron microscopy (HRTEM) image was recorded, and the corresponding fast Fourier transform (FFT) suggest Ag_2S along [210] direction.

Section S2: Raman mapping of the peak intensities after fabrication

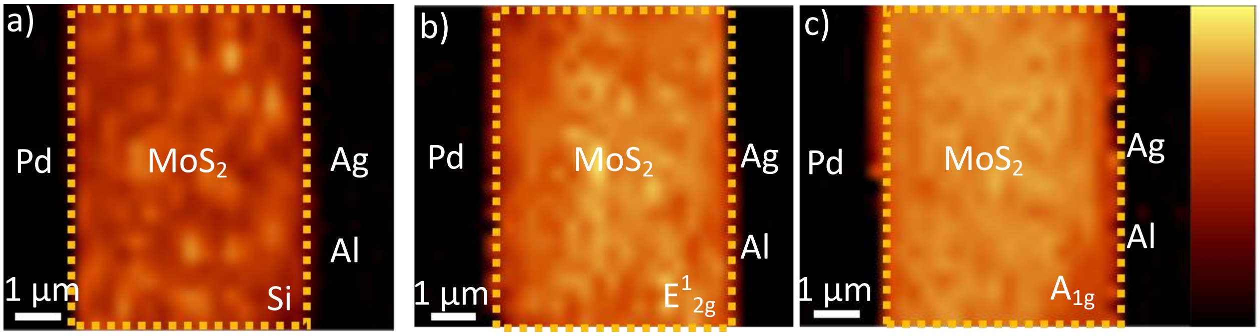

Figure S2: Raman map of the peak intensities after fabrication of the a) Si, b) E^1_{2g}, and c) A_{1g} peaks. The integrity of the molybdenum disulfide (MoS_2) channels after patterning by CF_4/O_2 reactive ion etching was verified via Raman mapping. The color scale increases to 30 CCD counts (higher – brighter).

Section S3: TEM characterization of MoS_2 after fabrication and electrical measurements

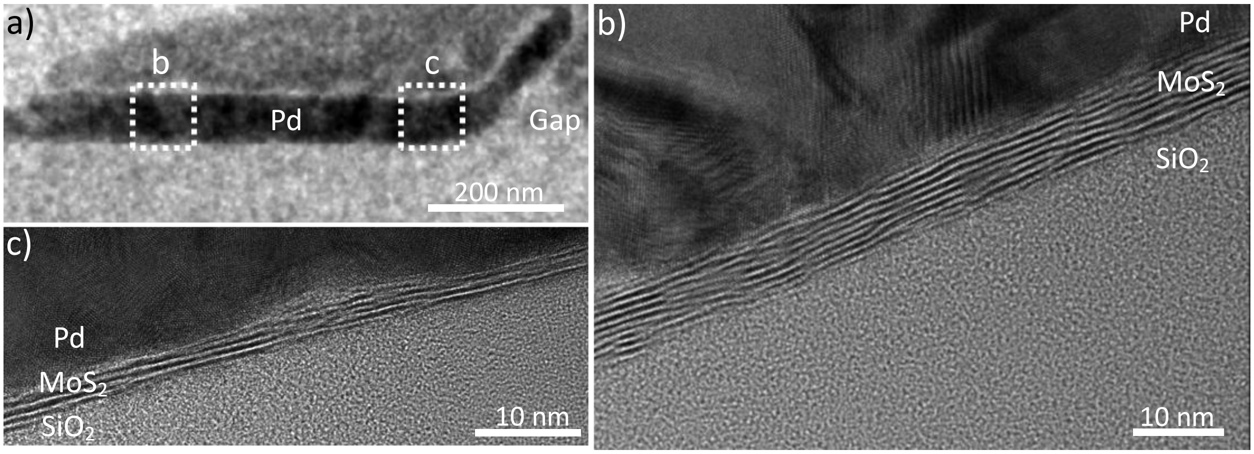

Figure S3: TEM and HRTEM cross-sectional images of MoS_2 after fabrication and electrical measurements. a) TEM cross-sectional image taken around the Pd electrode. Two different positions (b and c) along the Pd electrode are marked in white. b) HRTEM image of position b, which is located away from the gap between the metal electrodes. c) HRTEM image at position c, which is close to the gap between the metal electrodes. TEM imaging allows us to confirm the thickness variation of MoS_2 and the small difference in topography. Approaching the gap between the metal electrodes (position c), the MoS_2 layers were thinner, and more defects were observed. Close to the gap between the electrodes (position c), the number of layers measured was between 4 and 7, but 7 to 12 layers could be distinguished from it (position b). Some of this difference could be due to the wet transfer process, in which material can be left on the growth substrate.

Section S4: Raman spectra of as-grown MoS_2 on sapphire and after transfer

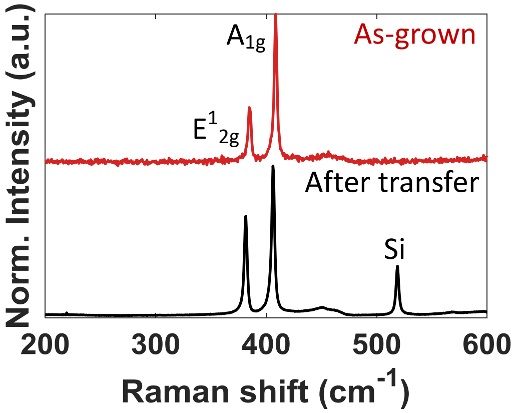

Figure S4: Raman spectra of as-grown MoS_2 on sapphire and after transfer on an SiO_2/Si substrate. Notably, the measurements were not taken at the exact same location, which could lead to a slight frequency difference due to thickness variation in the sample [1]. The extracted peaks for the as-grown MoS_2 on sapphire were 384.6 cm^{-1} and 408.5 cm^{-1} for the E^1_{2g} and A_{1g} peaks, respectively. These peaks coincide with those taken for more than four layers or bulk material. In the case of MoS_2 after transfer onto an SiO_2/Si substrate, the obtained values were 381.5 cm^{-1} and 406.1 cm^{-1} for the E^1_{2g} and A_{1g} peaks, respectively. These values match those previously reported for four layers or bulk [1,2].

Section S5: First switching cycles of forming-free devices

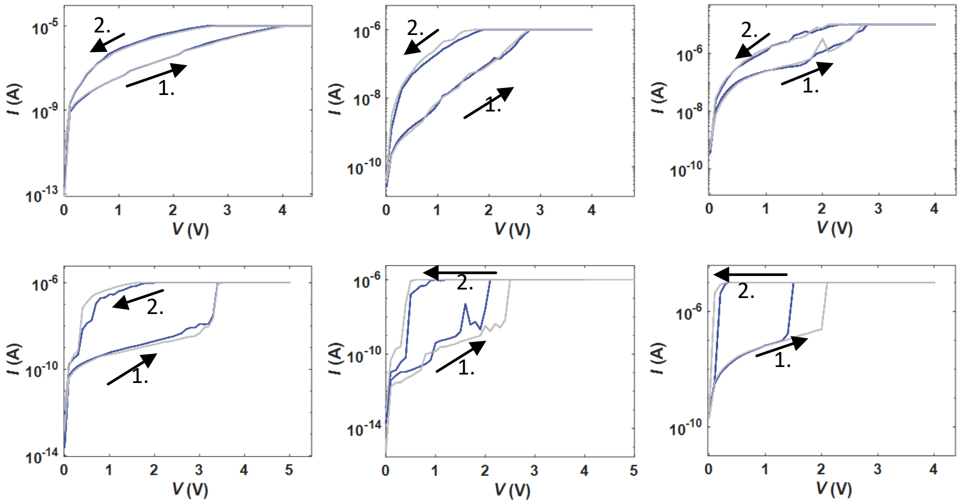

Figure S5: First switching cycles in logarithmic scale of several forming-free devices. Arrows 1 and 2 show the voltage sweep direction.

Section S6: *I-V* curves in logarithmic scale

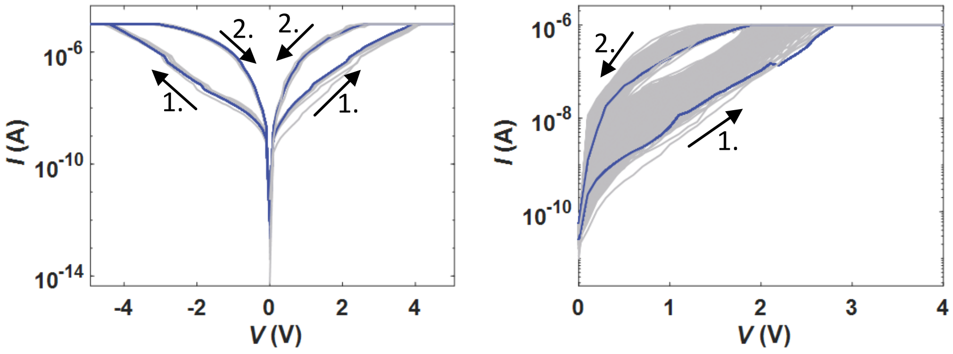

Figure S6: *I-V* curves from the manuscript in logarithmic scale. a) Ten subsequent voltage sweeps in both positive and negative polarities that show similar volatile RS behavior. The *I-V* sweeps were conducted first in the positive direction followed by the same procedure in the negative direction. Arrows 1 and 2 show the voltage sweep direction. The first switching cycle is marked in blue. b) 416 consecutive switching cycles on a device with a gap size of approximately 1.2 µm.

Section S7: Initial resistance states for different devices

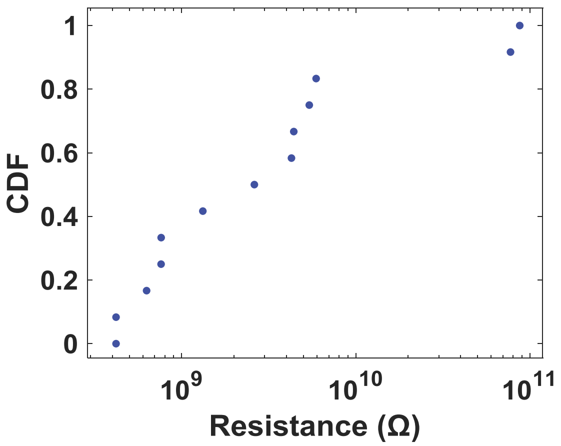

Figure S7: Cumulative distribution function (CDF) of the initial resistance states for 13 devices with different gap sizes. All the devices that were measured were initially in the high resistance state. Of the 13 devices, 4 of them required a forming process to enable RS.

Section S8: Forming *I-V* curves of different devices with large gap sizes

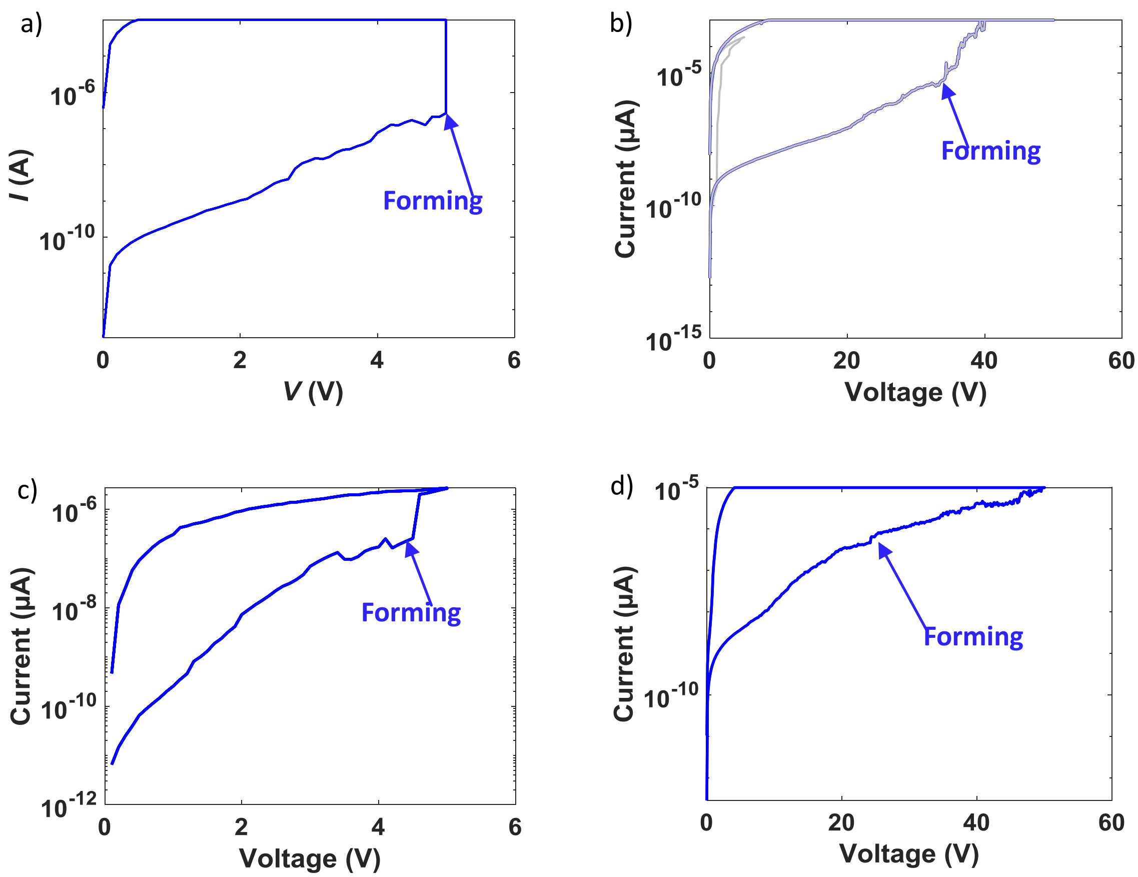

Figure S8: Forming *I-V* curves of the 4 devices from Figure S7. a) Forming of a device with a gap size of ~3.9 µm. b) Forming of a device with a gap size of ~5 µm. c) Forming of a device with a gap size of ~3.8 µm. d) Forming of a device with a gap size of ~5.8 µm.

Section S9: Higher current compliance for volatile RS

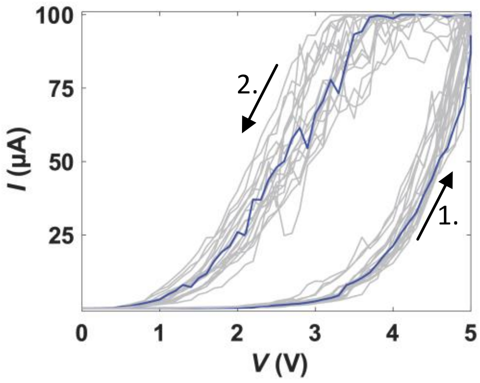

Figure S9: Twenty consecutive volatile RS cycles on a 1.2 μm device with 100 μA as the current compliance (CC). Arrows 1 and 2 show the voltage sweep direction.

Section S10: Proof of switching for different metal combinations with and without MoS_2

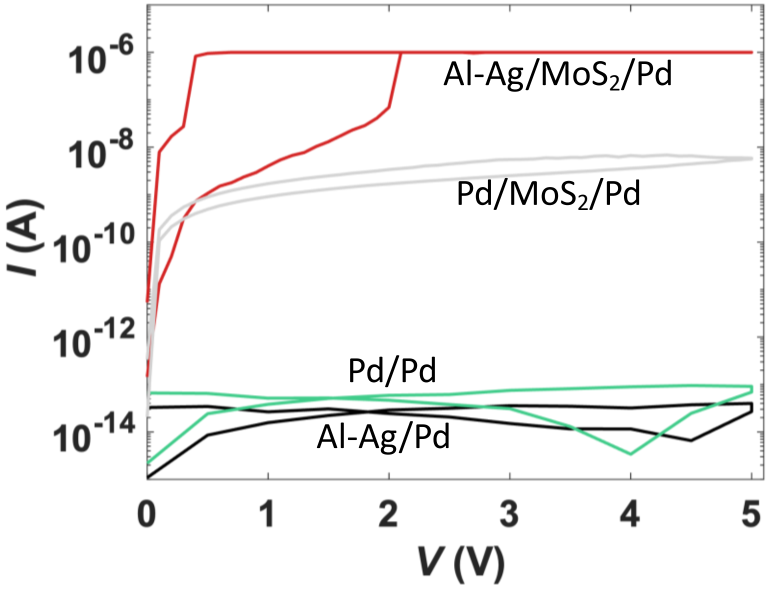

Figure S10: *Current–voltage* (*I–V*) sweeps of four devices with different metal combinations with and without MoS_2. These results also support that resistive switching (RS) originates from Ag ion migration on MoS_2. Pd/Pd- and Al-Ag/Pd-labeled measurements are electrodes where there is no MoS_2 bridging the gap.

Section S11: Histogram and Gaussian fit of the device data from Fig. 2b

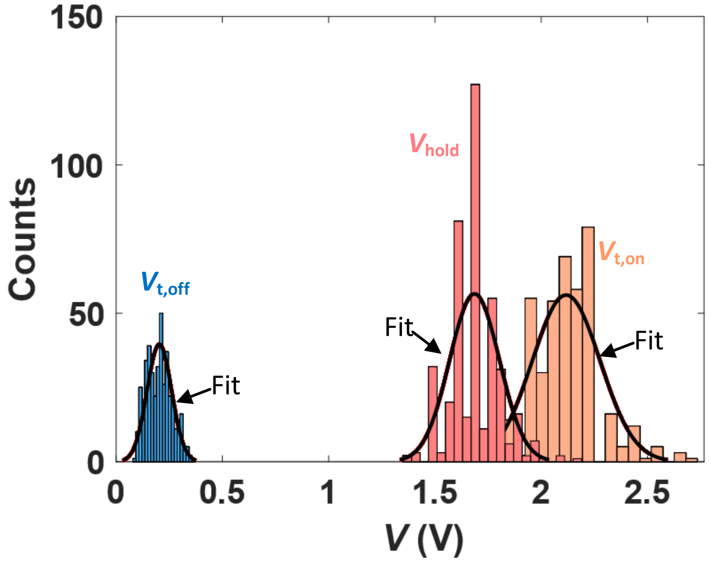

Figure S11: Histogram plot and Gaussian fit of $V_{t,on}$, V_{hold}, and $V_{t,off}$ extracted from endurance measurements with 416 subsequent *I*–*V* sweeps. The calculated means $V_{t,on}$, V_{hold}, and $V_{t,off}$ of 2.1 ± 0.1 V, 1.7 ± 0.1 V, and 0.2 ± 0.05 V, respectively, were extracted by fitting Gaussian distributions to the histogram data. A standard deviation of 0.1 V or lower indicates low cycle-to-cycle variability.

Section S12: Subsequent *I*–*V* sweeps for different gap sizes

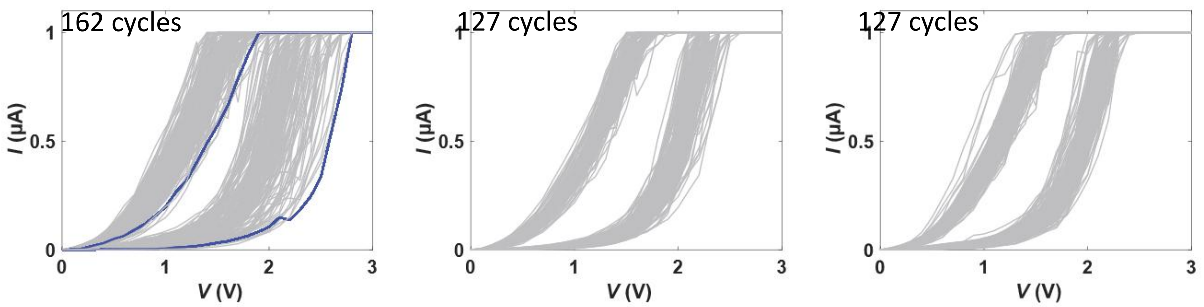

Figure S12-1: Raw data from 416 subsequent *I*–*V* sweeps from the device with a ~1.2 µm gap size. The first sweep is marked in blue.

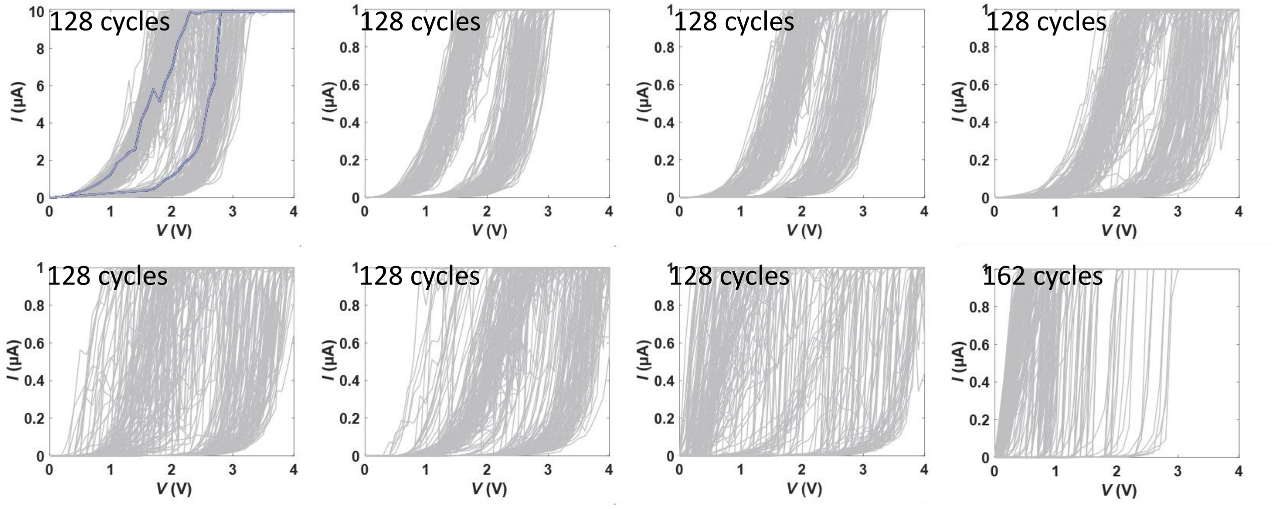

Figure S12-2: Raw data from 1058 subsequent *I*-*V* sweeps from the device with a ~2.1 μm gap size. The first sweep is marked in blue.

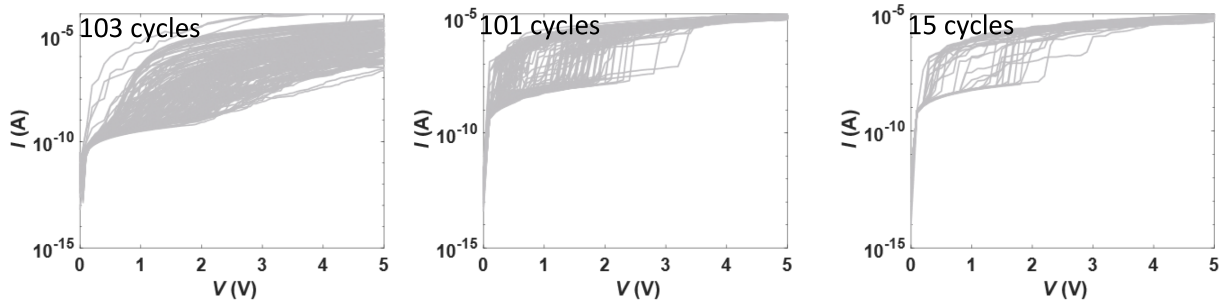

Figure S12-3: Raw data from 219 subsequent *I*–*V* sweeps from the device with a ~5.8 μm gap size. The forming step was not included in the plot.

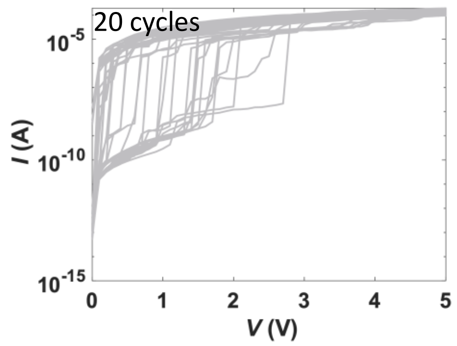

Figure S12-4: Raw data from 20 subsequent *I*–*V* sweeps from the device with a ~4.7 μm gap size. The forming step was not included in the plot.

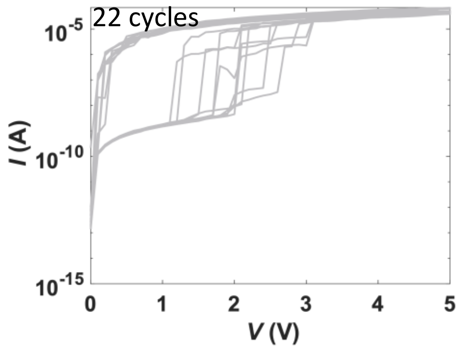

Figure S12-5: Raw data from 22 subsequent *I*–*V* sweeps from the device with a ~5.1 µm gap size. The forming step was not included in the plot.

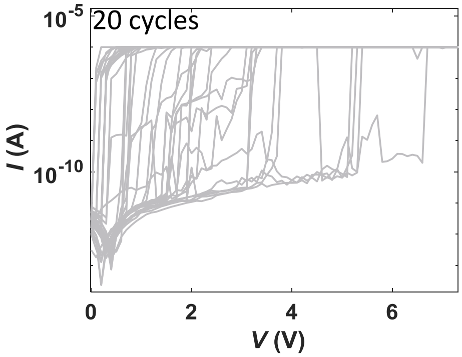

Figure S12-6: Raw data from 20 subsequent *I*–*V* sweeps from the device with a ~3.9 µm gap size. The forming step was not included in the plot.

Section S13: Dependence of $V_{t,on}$ upon the gap size

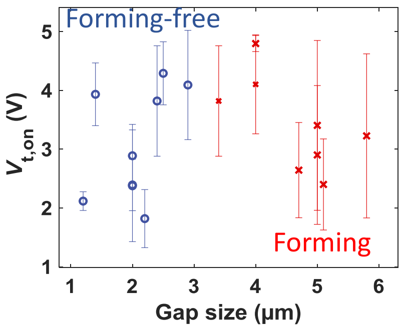

Figure S13: Dependence of $V_{t,on}$ upon the gap size for both with forming and forming-free devices. For each device at least 10 consecutive *I-V* sweeps were measured. The error bar was plotted using the standard deviation.

Section S14: Parameter extraction for pulse measurements

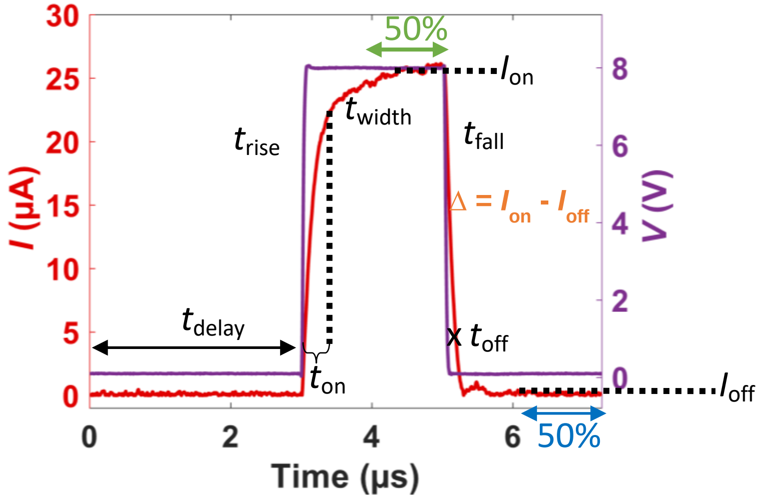

Figure S14: Pulsed waveforms with the following parameters were used: 3 µs delay time (t_{delay}), 50 ns rise and fall time (t_{rise} and t_{fall}), and 2 µs width (t_{width}).

Extraction and calculation of the on-switching characteristics:

1. Extrapolation of data, saving the new variables.

2. Consider the second half of the switching pulse width (marked in green).
3. Calculate the average of the current values in the green region to obtain I_{on}.
4. Calculate the 90% of the I_{on} and save this variable as $I_{on,90\%}$.
5. Find the corresponding index in x-axis and get the time for that current value.
6. Subtract t_{delay} from the obtained time in step 5 and save the new variable as t_{on}.
7. Calculate the average of the voltage values in the green region to obtain V_{on}.
8. The ON-state resistance was calculated with this formula: $R_{on} = V_{on} / I_{on}$.

Extraction and calculation of the off-switching characteristics:

1. Extrapolation of data, saving the new variables.
2. Consider the second half of the base pulse width (marked in blue).
3. Calculate the average of the current values in the blue region to obtain I_{off}.
4. Calculate the difference (delta, Δ) between the I_{on} and I_{off}.
5. Add the 10% of the delta to the I_{off}: $I_{off,delta} = I_{off} + 10\%\Delta$.
6. Find the corresponding index in x-axis and get the time for that current value.
7. Subtract (t_{delay}+ t_{rise}+ t_{width}) from the obtained time in step 6 and save the new variable as t_{off} (t_{relax}).
8. Calculate the average of the voltage values in the blue region to obtain V_{off}.
9. The OFF-state resistance was calculated with this formula: $R_{off} = V_{off} / I_{off}$.

Section S15: Comparison of device characteristics with those of other similar 2D-based memristive devices reported in the literature

Ref.	Structure	Growth	No. Layers	Gap length	Forming	Volatile	Cycles	$V_{t,on}$ (V)
**This work**	**Planar**	**MOCVD**	**7-12**	**1.2 µm**	**No**	**Yes**	**1058**	**~2**
[3]	Planar	CVD	1	2-7.5 µm	Yes	No	12	3.5-8.3
[4]	Planar	CVD	1	5–15 µm	Yes	No	475	~20
[5]	Planar	Exfoliated	5	10-40 nm	Yes	Yes	300	~0.4
[6]	Planar	Exfoliated	4	250 nm	Yes	No	1	~2
[7]	Planar	Exfoliated	~13	~4 µm	N/A	No	100	~10

Table S1: Benchmarking table of publications showing different characteristics of the devices.

Section S16: Pulse programming conditions for R_{on}, t_{on}, and t_{off} extraction

Voltage (V)	Rise	Width	Fall	Base Voltage (V)
2	1 µs	20 ms	1 µs	0.1
3	1 µs	3 ms	1 µs	0.1
4	50 ns	20 µs	50 ns	0.1
5	50 ns	5 µs	50 ns	0.1

Table S2: Pulse programming conditions for R_{on}, t_{on}, and t_{off} extraction.

Section S17: First-principles calculations of Ag adsorption on the MoS_2 surface

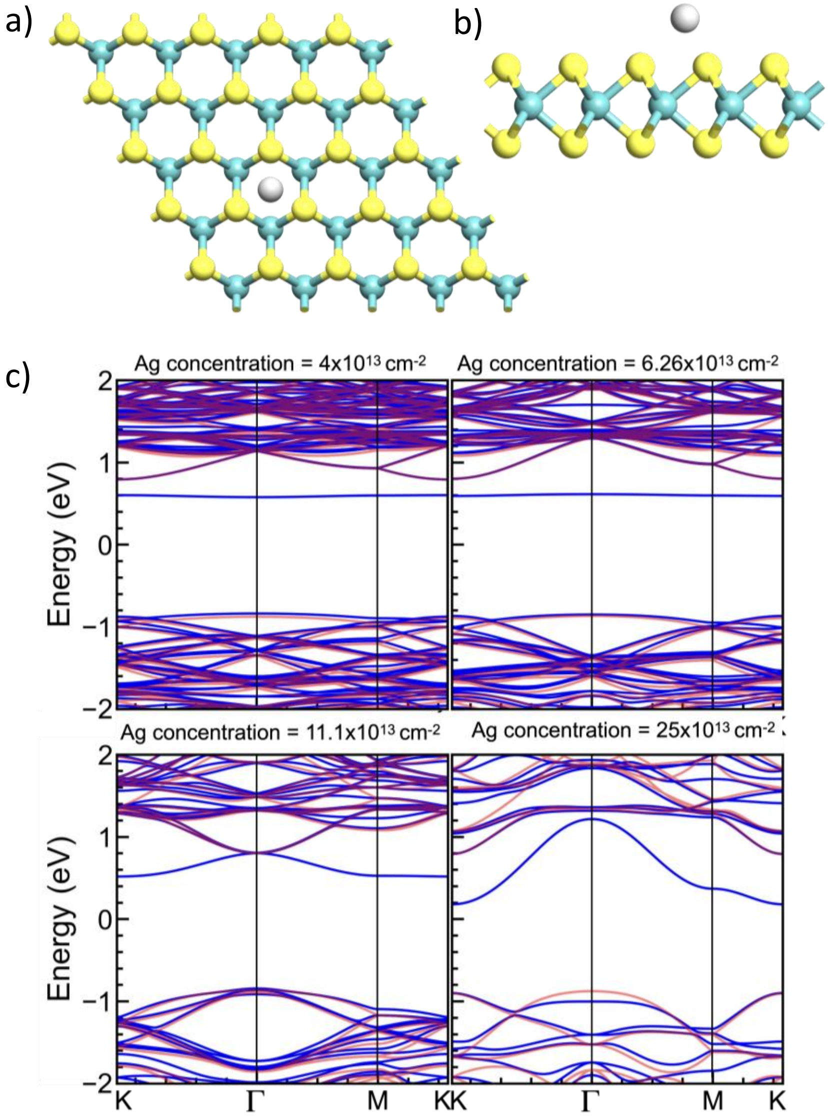

Figure S15: a) Top view and b) side view of monolayer MoS_2 supercell with surface-adsorbed Ag and c) band structure calculated via density functional theory (DFT) (solid blue) for surface-adsorbed Ag with gradually increasing concentration, showing the transition of the localized to delocalized gap state. The band structure of pristine MoS_2 is also shown in semitransparent red for comparison.

Section S18: Physical modeling of the volatile resistive switching (RS) memristor

According to the *ab initio* results, the presence of Ag atoms along the MoS_2 channel and their concentration determine the electronic transport mechanism in the memristive device. Figure S10 shows a schematic illustration of the analyzed structure, where *L* is the distance between the Pd and Ag contacts and *d* represents the average distance among Ag ions.

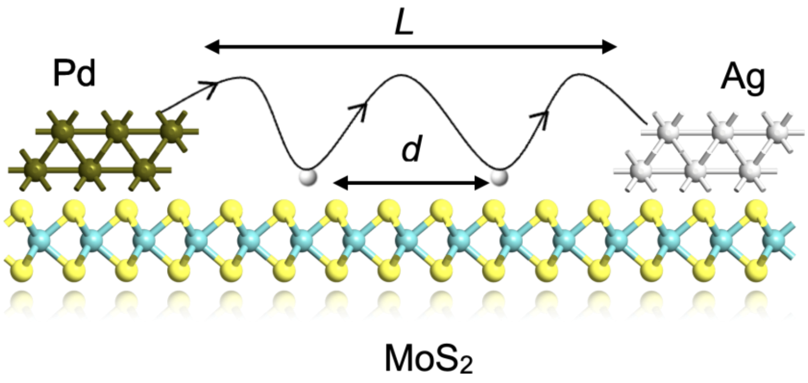

Figure S16: Schematic showing the monolayer MoS_2 with surface-adsorbed Ag, where *L* is the gap length and d is the average distance between adjacent Ag atoms.

As discussed in the main text, the I–V characteristics of the HRS and LRS can be attributed to hopping and space-charge-limited conduction (SCLC) transport, respectively.

The complete memristor model is developed in two stages: i) first, the movement of the Ag ions within the device is considered, and ii) this Ag dynamics is connected to the current conduction mechanism via electron transport.

To this end, the movement of the Ag ions is modeled on the basis of the probability of the ions overcoming the migration barrier according to the Arrhenius law. The average distance between adjacent Ag ions (*d*) is considered the state variable, defining the dynamics of the resistance state. Following, ref.[8] The time derivative of *d* can be written as

$$\frac{d}{dt}(d) = -V_0 exp\left(-\frac{E_a}{kT}\right) sinh\left(\gamma \frac{qA_0}{kT}\frac{V - SHIFT}{L}\right)$$

$$\gamma = \gamma_0 - \beta d^3$$

where E_a is the migration barrier for Ag ions; *k* is the Boltzmann constant; *T* is the absolute temperature; *q* is the elementary charge; *L* is the gap length (distance between two contacts); and V_0, A_0, SHIFT, γ_0 and β are fitting parameters. Here, the introduction of the parameter SHIFT ensures that the memristor resets when the voltage value crosses the off voltage ($V_{t,off}$), capturing the volatile nature of the memristor.

The value of *d* determines the conduction state of the memristor, as it depends on the history of the applied voltage. It is bounded in the model implementation to avoid the inclusion of unphysical values. The current conduction due to electron transport is then modeled as follows:

From Ohms' law, the electron current in the memristor can be written as

$$I = nq\mu A\frac{V}{L}$$

where *n* is the electron density, *A* is the cross-sectional area and μ is the mobility. In the case of disordered systems, the mobility due to hopping is given by [9]:

$$\mu = \mu_0 \exp\left(-\frac{E_{act}}{kT} + \frac{G_0}{kT}\sqrt{\frac{V}{L}}\right)$$

where μ_0 is the field-independent mobility, E_{act} is the activation energy for electron hopping and G_0 is the field enhancement factor, which is used here as a fitting parameter.

With this definition of μ, the current can be written as

$$I = I_0 \cdot n \cdot exp\left(\frac{G_0}{kT}\sqrt{\frac{V}{L}}\right) \cdot \frac{V}{L}$$

$$I_0 = q\mu_0 A exp\left(-\frac{E_{act}}{kT}\right)$$

In addition to the carrier hopping assisted by the applied field captured through the μ, the distance between the Ag ions is also dynamically modified with the applied bias. This modification affects the band structure of MoS_2, as confirmed via DFT calculations (Fig. S10). This change in the band structure can be captured through the electron density (*n*), which can be modeled on the basis of *d* as follows:

Case 1: When d → 0, a delocalized band appears in the bandgap, giving rise to band transport, and the electron density in such a case (n_0) can be written via the Gauss law as

$$qn_0L = \epsilon_s \frac{V}{L}$$

$$\therefore n_0 = \frac{\epsilon_s}{q}\frac{V}{L^2}$$

where ϵ_s is the permittivity of MoS_2.

Case 2: When *d* → *L*, the electron density is equal to the number of electrons at the localized trap state (n_t)

Therefore, at any intermediate *d*, the value of *n* will be the addition of the trap carrier n_t and the fraction of n_0 that can jump overcoming the barrier, which is proportional to the jump rate $exp(-\alpha d)$ [10]. The total *n*, therefore, is modeled as

$$n = \left(\frac{n_0 exp(-\alpha d) + n_t}{1 + exp(-\alpha d)}\right)$$

Using the derived value of *n*, the total electron current through the memristor can be written as

$$I = I_0 \left[\frac{\frac{\epsilon_s V}{qL^2} exp(-\alpha d) + n_t}{1 + exp(-\alpha d)} \right] exp\left(\frac{G_0}{kT} \sqrt{\frac{V}{L}} \right) \frac{V}{L}$$

The expression for the current can be simplified in the following scenarios:

1: when *d* is large,

$$I = I_0 n_t exp\left(\frac{G_0}{kT} \sqrt{\frac{V}{L}} \right) \frac{V}{L}$$

which describes hopping transport with field-enhanced mobility [9] or Poole-Frenkel (PF)-type transport that follows the experimentally observed behavior in the HRS, i.e., $\frac{I}{V} \propto exp(\sqrt{V})$.

2: when *d* is small,

$$I = I_0 \frac{\epsilon_s}{q} \exp\left(\frac{G_0}{kT} \sqrt{\frac{V}{L}} \right) \frac{V^2}{L^3}$$

which describes the SCLC with PF-enhanced mobility [11] and follows the experimentally observed behavior in the LRS.

Therefore, the model proposed here efficiently captures the switching mechanism as the carrier transport changes from hopping to SCLC via a single continuous equation.

Section S19: Metal-organic chemical vapor deposition (MOCVD) of MoS_2

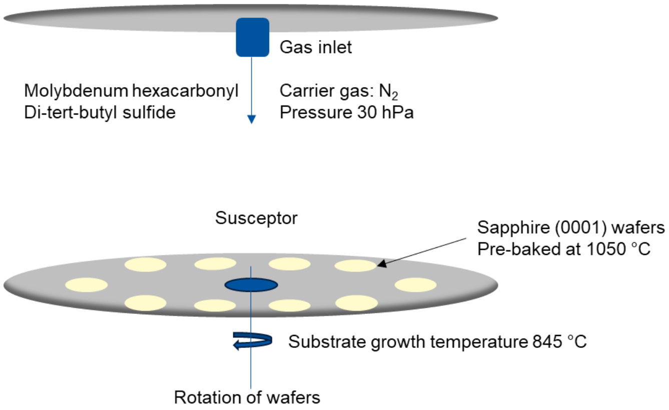

Figure S17: Schematic of the MOCVD growth of MoS_2 on 2" sapphire. MoS_2 was epitaxially grown in a commercial AIXTRON planetary reactor in a 10×2" configuration on sapphire (0001) substrates. The substrates were prebaked at 1050 °C in a pure H_2 atmosphere [12]. The growth process was carried out at a substrate temperature of 845 °C, with nitrogen as the carrier gas and a pressure of 30 hPa. A high sulfur to molybdenum ratio of 200000 was chosen to achieve homogeneous MoS_2 films on a wafer scale.

Section S20: Flowchart of the fabrication process of lateral devices

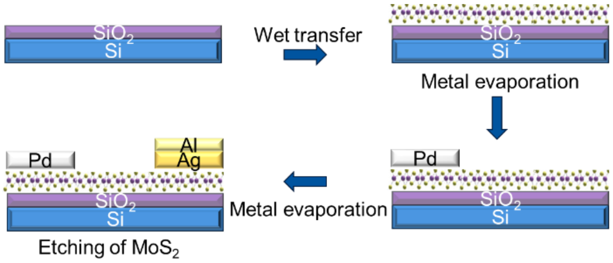

Figure S18: Schematic process flow for device fabrication. MoS_2 was transferred onto 2 x 2 cm^2 Si chips covered with 275 nm thermal SiO_2. The asymmetric Pd (50 nm) and Ag (50 nm)/Al (50 nm) electrodes were deposited via electron-beam evaporation Finally, the channels were patterned via CF_4/O_2 reactive ion etching (RIE).

References

[1] C. Lee, H. Yan, L. E. Brus, T. F. Heinz, J. Hone, S. Ryu, *ACS Nano* **2010**, *4*, 2695.

[2] H. Li, Q. Zhang, C. C. R. Yap, B. K. Tay, T. H. T. Edwin, A. Olivier, D. Baillargeat, *Adv Funct Materials* **2012**, *22*, 1385.

[3] V. K. Sangwan, D. Jariwala, I. S. Kim, K.-S. Chen, T. J. Marks, L. J. Lauhon, M. C. Hersam, *Nature Nanotech* **2015**, *10*, 403.

[4] V. K. Sangwan, H.-S. Lee, H. Bergeron, I. Balla, M. E. Beck, K.-S. Chen, M. C. Hersam, *Nature* **2018**, *554*, 500.

[5] M. Farronato, M. Melegari, S. Ricci, S. Hashemkhani, A. Bricalli, D. Ielmini, *Adv Elect Materials* **2022**, *8*, 2101161.

[6] S. Yin, Z. Luo, Q. Li, C. Xiong, Y. Liu, R. Singh, F. Zeng, Y. Zhong, X. Zhang, *Phys. Status Solidi A* **2019**, *216*, 1900104.

[7] G. Ding, B. Yang, R.-S. Chen, W.-A. Mo, K. Zhuo, Y. Liu, G. Shang, Y. Zhai, S.-T. Han, Y. Zhou, *Small* **2021**, *17*, 2103175.

[8] Z. Jiang, Y. Wu, S. Yu, L. Yang, K. Song, Z. Karim, H.-S. P. Wong, *IEEE Trans. Electron Devices* **2016**, *63*, 1884.

[9] I. I. Fishchuk, A. Kadashchuk, M. Ullah, H. Sitter, A. Pivrikas, J. Genoe, H. Bässler, *Phys. Rev. B* **2012**, *86*, 045207.

[10] M. Van Der Auweraer, F. C. De Schryver, P. M. Borsenberger, H. Bässler, *Advanced Materials* **1994**, *6*, 199.

[11] P. N. Murgatroyd, *J. Phys. D: Appl. Phys.* **1970**, *3*, 151.

[12] M. Marx, S. Nordmann, J. Knoch, C. Franzen, C. Stampfer, D. Andrzejewski, T. Kümmell, G. Bacher, M. Heuken, H. Kalisch, A. Vescan, *Journal of Crystal Growth* **2017**, *464*, 100.
